# Annual changes in the Biodiversity Intactness Index in tropical and subtropical forest biomes, 2001–2012

**DOI:** 10.1038/s41598-021-98811-1

**Published:** 2021-10-12

**Authors:** Adriana De Palma, Andrew Hoskins, Ricardo E. Gonzalez, Luca Börger, Tim Newbold, Katia Sanchez-Ortiz, Simon Ferrier, Andy Purvis

**Affiliations:** 1 Department of Life Sciences, Natural History Museum, London, SW7 5BD UK; 2 CSIRO Land and Water, Canberra, ACT Australia; 3 CSIRO Health and Biosecurity, Townsville, Qld Australia; 4 Department of Life Sciences, Imperial College London, Ascot, SL5 7PY UK; 5 Department of Biosciences, University of Swansea, Swansea, SA2 8PP UK; 6 Department of Genetics, Evolution and Environment, Centre for Biodiversity and Environment Research, University College London, Gower Street, London, WC1E 6BT UK

**Keywords:** Biodiversity, Ecological modelling

## Abstract

Few biodiversity indicators are available that reflect the state of broad-sense biodiversity—rather than of particular taxa—at fine spatial and temporal resolution. One such indicator, the Biodiversity Intactness Index (BII), estimates how the average abundance of the native terrestrial species in a region compares with their abundances in the absence of pronounced human impacts. We produced annual maps of modelled BII at 30-arc-second resolution (roughly 1 km at the equator) across tropical and subtropical forested biomes, by combining annual data on land use, human population density and road networks, and statistical models of how these variables affect overall abundance and compositional similarity of plants, fungi, invertebrates and vertebrates. Across tropical and subtropical biomes, BII fell by an average of 1.9 percentage points between 2001 and 2012, with 81 countries seeing an average reduction and 43 an average increase; the extent of primary forest fell by 3.9% over the same period. We did not find strong relationships between changes in BII and countries’ rates of economic growth over the same period; however, limitations in mapping BII in plantation forests may hinder our ability to identify these relationships. This is the first time temporal change in BII has been estimated across such a large region.

## Introduction

Biodiversity indicators can play an essential role in tracking progress towards policy targets, especially if the indicators link strongly to both the targets and biodiversity, have broad geographic coverage, and are available as a time series^1^. These stringent criteria, together with the pronounced geographic biases in biodiversity data availability^2–4^, have contributed to a strong taxonomic bias in global biodiversity indicators^1,5–7^. In an assessment of whether the rate of biodiversity loss had fallen by 2010^7^, only one of four measures of the state of biodiversity considered any non-vertebrate data (the Red List Index considered corals in addition to birds, mammals and amphibians) and none of the three indicators of benefits accrued from biodiversity did so. This bias is, if anything, stronger among indicators considered in a mid-term analysis of progress towards the Aichi 2020 Targets^1^: only one of the nine measures of the state of biodiversity (coral reef cover) considered non-vertebrate data, and none of the three measures of benefits did so. Indicators based on a taxonomically-broad sets of species are urgently needed^8^ because species in different clades often respond differently to given human activities^9–12^. Given the current state of biodiversity data availability, models offer the best immediate prospect for estimating biodiversity indicators with broad taxonomic coverage and good spatial and temporal resolution worldwide^13,14^. Despite the growth of databases that collate time-series data for populations^15^ and assemblages^16^, such data are as yet too sparse to produce fine-grained estimates of rates of change through data aggregation^14,17^. Additionally, geographic biases in the sites from which such data are available mean that the average trends they show may not accurately reflect the true global trend^18–20^.

The Biodiversity Intactness Index (BII) is a model-based indicator of terrestrial biodiversity that has been designed to allow broad taxonomic coverage and good spatiotemporal resolution^21^. BII is defined as ‘the average abundance of a large and diverse set of organisms in a given geographical area, relative to their reference populations’, with the reference condition approximated by the contemporary situation in minimally-impacted sites, given the paucity of sufficiently precise historical baseline data^21^; it is therefore a measure of the average state of local biodiversity. Although human impacts on the rate of global species extinction have perhaps attracted more concern^22–24^, local diversity matters more than global diversity for reliable provision of many ecological functions and services. Reduction in local diversity is associated with reduced rates of delivery of key functions^25^ as well as greater variance in those rates^26^. This closer link to function is one reason why the Biodiversity Intactness Index (BII) was proposed as a metric that could be used to assess the state of biodiversity relative to its proposed ‘Planetary Boundary’—i.e., the boundary beyond which if biodiversity continues to decline, Earth System functioning may suffer^27,28^. In addition, losses across trophic groups can have larger impacts on ecosystem function than losses within a trophic group^26^ so, by including multiple taxa, BII may be more functionally relevant than many other measures of local diversity. BII usefully complements indicators focusing on species populations or extinction^29^. Additionally, BII combines aspects of both alpha diversity (total abundance) and beta diversity (compositional similarity) to estimate the average local abundance of naturally-present species. These two aspects of diversity can show contrasting patterns^30^ and responses to human impacts^31,32^. The contrast may in part help to resolve the recent debate on how human impacts have been affecting the diversity of local ecological assemblages^33^.

BII can be estimated by combining statistical models of how land use (the main driver of terrestrial biodiversity loss^34^) and related anthropogenic pressures (e.g., land-use intensity and human population density) affect assemblages^19^ with global maps of these factors. We previously^35^ used this approach to estimate BII globally for the year 2005 using assemblage data from the PREDICTS database^36^, a large global compilation of primary studies that compared ecological assemblages at sites facing different land-use pressures.

Here we use annual global fine-resolution maps of land use and human population density to map modelled BII at 30-arc-second resolution ($\sim 1$ km at the equator) across the world’s tropical and subtropical forest biomes for each year from 2001 to 2012. These biomes are generally under-represented in global biodiversity^16,20^—presenting a real challenge for producing well-resolved biodiversity indicators through data aggregation—but are home to most of the world’s terrestrial species, provide ecosystem services that sustain well over one billion people^37^, and face severe anthropogenic threats, especially in south east Asia^38,39^. The main current threat to tropical forest biodiversity is land-use change^40^, driven by a combination of factors that include agricultural expansion, timber extraction and infrastructure development^41^, with rates and patterns of forest loss differing regionally^37^. Deforestation and degradation reduce local species richness across a range of groups^12,42,43^ but no biodiversity indicators are yet available that give a taxonomically broad picture. Model-based indicators such as BII are able to take advantage of developments in remote sensing that have greatly improved the ability to track land-cover change—particularly forest loss^44^—at fine spatial and temporal resolutions^45^. Direct exploitation is also a major threat in these biomes, its intensity related to both human population density and accessibility^46^; our statistical models therefore also include both human population density and road density.

Estimating BII for each year greatly enhances its usefulness as an indicator. We summarise changes in average BII at national and regional levels to facilitate biodiversity assessments such as those undertaken by the Intergovernmental Science-Policy Platform on Biodiversity and Ecosystem Services (IPBES) and the Group on Earth Observations (GEO). We also explore how per capita Gross Domestic Product (GDP) is related to average change in BII across countries. The relationship between economic indicators and biodiversity is still unclear. Recent evidence has suggested that increases in GDP are correlated with increased forest area in tropical forested regions^47^, while forest degradation and loss is concentrated in poorer countries^48^; however, other work has shown that increased GDP is correlated with increased forest change^49^ and extinction risk in mammals^50^.

## Results

In the mixed-effects models that underpin the estimation of BII, land use, land-use intensity, human population density and road density were all highly significant influences on ecological assemblages. The model of compositional similarity between pairs of sites in the PREDICTS database (i.e., the asymmetric Jaccard’s similarity of species abundances between a baseline site—minimally-used primary vegetation—and another site in the same study), could not be simplified, as the factor combining land use and land-use intensity interacted significantly with the three covariates (human population density, road density at the 50 km scale and road density at the 1 km scale: all $p < 0.01$ according to permuted likelihood ratio tests). In the model of total site-level abundance of organisms (i.e., at a site, the sum of abundance of all species sampled), the land-use factor interacted significantly with both human population density ($\chi ^2 = 22.23$, df = 10, $p < 0.05$) and road density at the 50km scale $\chi ^2 = 25.10$, df = 10, $p < 0.01$). Road density at 1 km was not maintained in the model for total abundance. The R^2^ values for the models of compositional similarity and total abundance were 0.56 and 0.67 respectively^51,52^. See the “Supplementary Material” for additional R^2^ values and predictive performance.

Human dominated land uses often had significantly lower diversity than in minimally-used primary vegetation (when all other variables were set to mean levels), particularly in terms of compositional similarity (See the “Supplementary Material” for full model coefficients). However, even in primary vegetation, broader-scale pressures led to declines in diversity. As road density at the 50 km scale increases, abundance tended to decline in minimally-used primary vegetation, but the trend was not significant (estimate (est) = $-0.0283$, standard error (se) = 0.02, t-value (t) = $-1.81$, lower bootstrapped Confidence Interval (bCI) = $-0.06$, upper bCI = 0.00). Compared to this trend, however, abundance declined significantly more rapidly in lightly-used primary vegetation as road density increased (est = $-0.0419$, se = 0.02, t = $-2.55$, lower bCI = $-0.0744$, upper bCI =  $-0.0083$). Road density at the 50km scale also resulted in some of the most extreme declines in compositional similarity, particularly in lightly and intensively-used primary vegetation. In minimally-used primary vegetation, there was a significantly negative relationship between compositional similarity and road density (est = $-1.624$, se = 0.02, t = $-8.16$, $p < 0.001$); this relationship was significantly stronger in lightly and intensively-used primary vegetation (est = $-0.1694$, se = 0.05, t = $-3.66$, $p < 0.001$) where declines in abundance with increasing road density were much more rapid.

Human population density on the other hand seemed to have the strongest marginal impact on biodiversity in secondary vegetation sites. In minimally-used primary vegetation, abundance did not change significantly as human population density increased (est = 0.0141, se = 0.01, t = 1.11, lower bCI = $-0.01$, upper bCI = 0.04). Compared to this trend, abundance declined much more rapidly with increasing human population density in minimally- (est = $-0.0422$, se = 0.02, t = $-2.57$, lower bCI = $-0.0751$, upper bCI = $-0.0086$) and lightly-used (est = $-0.0362$, se = 0.02, t = $-2.1$, lower bCI = $-0.0726$, upper bCI = $-0.0025$) secondary vegetation. As human population density increased, compositional similarity declined significantly in minimally-used primary vegetation (est = 0.3349, se = 0.04, t = 9.45, $p < 0.001$); compared to this trend, compositional similarity declined even more steeply in secondary vegetation when human population density increased (est = $-0.3403$, se = 0.03, t-value = $-11.53$, $p < 0.001$).

**Figure 1:**
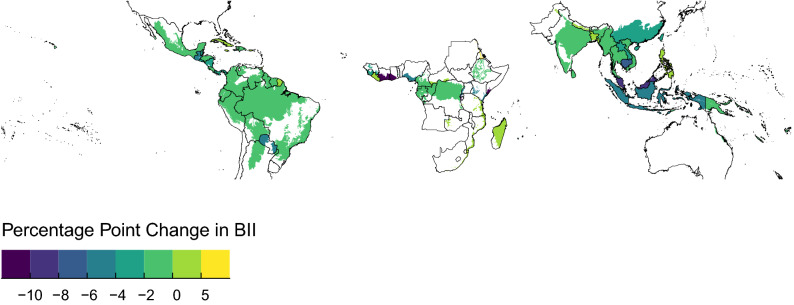
Map of country level differences in BII between 2001 and 2012 (expressed as percentage point difference). Increases in BII can occur if the abundance of originally-present species increases, so there is no upper limit to BII values and therefore to possible percentage point increase. BII cannot be less than zero, therefore the maximum possible decrease in BII is $-95$ percentage points (as the highest country-level mean value of BII in 2001 was  95%).

On average across the tropical and subtropical forested biomes, BII was 61.7% in 2012, with Bangladesh, Haiti and India having particularly low values while Suriname, Papua New Guinea and French Guiana still had values exceeding 90% (Fig. 1). The average BII across the three biomes assessed was 63.6% in 2001, meaning the rate of loss has been approximately 0.17% per year on average, but losses have varied geographically (Fig. 1 and Appendix Fig. 1). BII fell most rapidly in the Tropical and Subtropical Moist Broadleaf Forests (with a loss of 2.03 percentage points from 2001 to 2012), but remained relatively stable at 50.5% in Tropical and Subtropical Coniferous Forests. All regions saw an average decline in BII over the period, with Asia and the Pacific suffering the greatest losses ($-2.3$ percentage points) and the least severe declines in the Americas ($-1.63$ percentage points); similarly, Asia and the Pacific suffered the sharpest declines in the area of primary vegetation ($-6.0$% relative to the area in 2001), while Africa saw a minor average increase ($+3$%).

**Figure 2:**
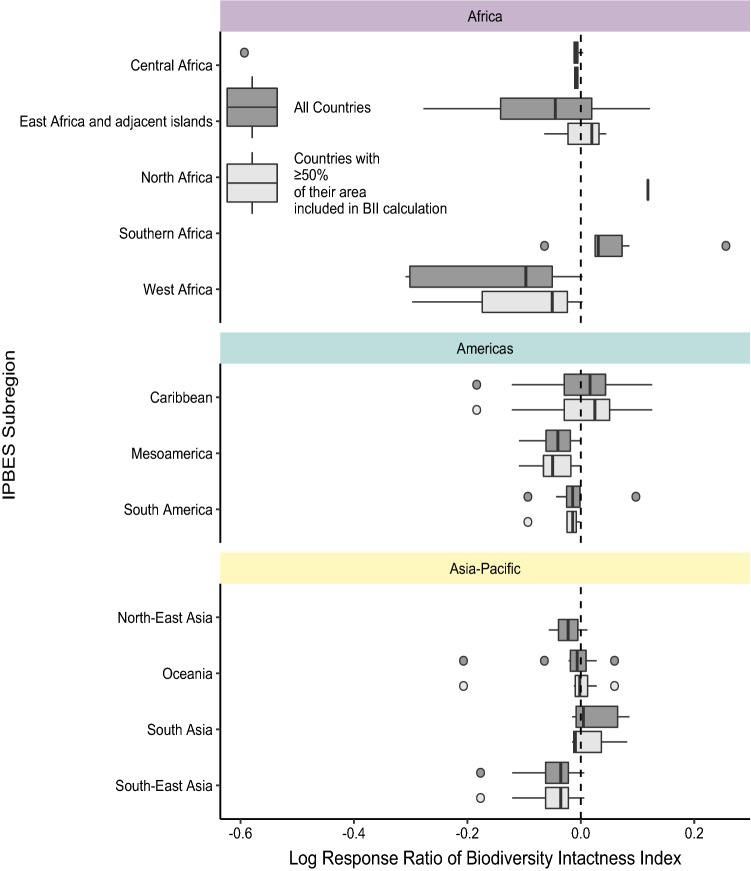
Average change in BII over time at the country level, across different subregions. Change was calculated as the log-response ratio of 2012 and 2001 values. A value of zero indicates no change (identified by the dashed line), negative values indicate a decline over time, and positive values indicate an increase in BII over time. Darker boxes include all countries; lighter boxes use data for countries where BII has been calculated for at least 50% of their area. The center line of the boxplot indicates the median value, boxes show data within the 25th–75th percentiles, whiskers show points that are up to $1.5 \times$ the interquartile range of the data, points are data that fall outside of these limits. Outliers are shown as points.

Changes in BII from 2001 to 2012 varied among countries (Fig. 2), but the median log response ratio was significantly negative (median = $-0.01$, Wilcoxon test: V = 2814, $p < 0.01$). When considering only those countries where at least 50% of their area is within the included tropical or subtropical forest biomes, the results remained qualitatively similar, with most countries showing average losses over the time period (Fig. 2).

**Figure 3:**
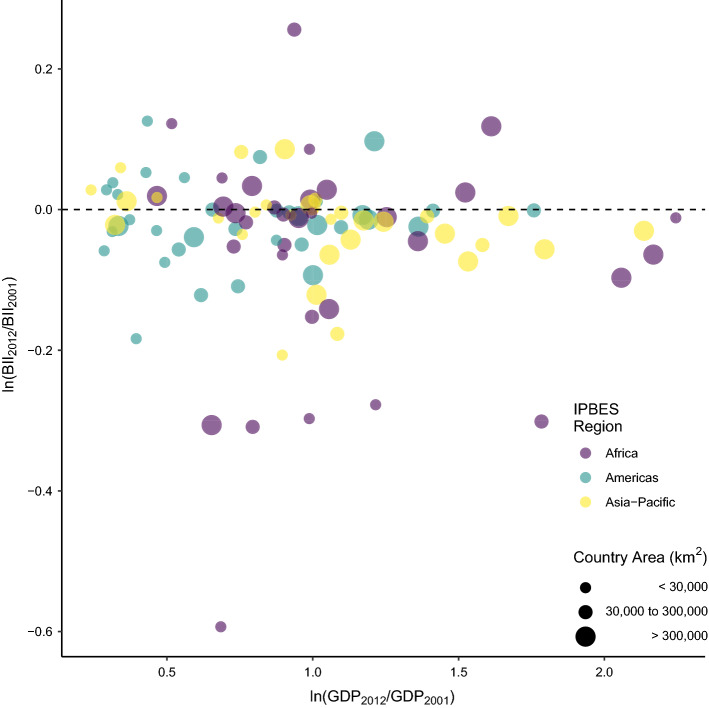
Change in BII over time plotted against the change in Gross Domestic Product (GDP) per capita for each country. Change was calculated as the log-response ratio of 2012 and 2001 values. A value of zero indicates no change, negative values indicate a decline from 2001 to 2012, and positive values indicate an increase between 2001 and 2012. Note that not all countries have available data on GDP per capita for the years 2001 and 2012 so some countries are excluded from this plot. Colours represent the different regions. The circles are scaled according to the country area.

Average change over time at the country level was not clearly related to changes in GDP per capita (estimate = $-0.0182$, se = 0.03, t-value = $-0.71$, $p= 0.48$; Fig. 3) or to GDP in 2001 (estimate = 0.03, se = 0.02, t-value = 1.77, $p = 0.08$).

## Discussion

Our estimate of average BII across tropical and subtropical forest biomes for the year 2012 (61.7%) is far below the precautionary ‘safe limit’ of Planetary Boundary for biosphere integrity, set by Steffen et al.^28^ at a value of 90%. The Planetary Boundaries framework argues that transgressing safe limits risks perturbing natural negative feedbacks that have maintained the earth system in a stable state throughout the history of civilisation^28,53^. In that framework, BII is intended to reflect the ability of ecological assemblages to reliably provide ecosystem function and services on which society depends, with the ‘safe limit’ representing a threshold below which positive feedbacks make severe large-scale disruption to ecosystem services increasingly likely^27^. It is unclear whether the earth system really has such a biophysical threshold for biodiversity integrity—either globally or across particular biomes as analysed here—or what value it takes if so^27,54–56^ (which Steffen et al.^28^ reflected by proposing the true safe limit lies somewhere in the range 30–90%). However, even without a biophysical threshold, worsening socioeconomic disruption is a predictable consequence of large-scale scarcity of goods and services^57^, which is in turn a predictable consequence of unsustainable use of ecological resources^55^. The low global value and ongoing rapid loss of BII therefore present a worrying picture of the state of biodiversity in tropical and subtropical forest biomes.

BII is of course far from the first evidence of the severity of the state of biodiversity in these biomes. Meta-analyses have shown the impact of forest degradation, fragmentation and loss on biodiversity^42,58,59^. Tropical population time series show the ongoing steep decline^2^; and a composite index based on carefully-structured expert judgement shows widespread decline even in protected areas^60^. However, as a model-based indicator based on a large and taxonomically representative database^19,36^, BII can be estimated at high spatiotemporal resolution across the whole set of biomes, making it possible to explore geographic variation in status and trends.

Only three countries are estimated to have remained above the 90% BII threshold: French Guiana, Suriname and Papua New Guinea. This is in part because these countries maintained high levels of primary vegetation and low levels of urbanisation and human population density throughout the time period assessed. Most countries (81 of 124) and all but three subregions showed a decline in average BII from 2001 to 2012, with South East Asia seeing particularly widespread rapid losses^61^. However, it is important to note that most changes in BII are relatively small (From 2001 to 2012, 98 of 124 countries changed BII by between $-5$ and 5 percentage points; See Appendix Fig. 1). The largest decrease in BII was in Côte d’Ivoire and appeared to be a result of agricultural expansion, as secondary vegetation decreased while the area of cropland increased. Agricultural abandonment, however, appears to have driven the largest inferred increase in BII, in the Cocos (Keeling) Islands. This inference is likely to be overly optimistic, however, as it will take time to fully achieve these gains and we do not yet include the temporal dynamics of recovery in these models (see limitations below).

The statistical models underpinning the estimation of BII highlight that land-use conversion was of course an important predictor of biodiversity loss, but degradation at local (1 km) and broader (50 km) scales were also significant contributors: for primary vegetation, some of the strongest declines in compositional similarity were seen as road density increased at broader spatial scales, particularly in lightly and intensively-used primary vegetation. This finding suggests that natural intact vegetation must be protected at varying spatial scales in order to conserve local diversity^62^. Indeed, the amount of natural intact vegetation is low and continuing to decline^63–65^, and the rapid losses in south east Asia reflect the rapid recent loss of natural forest across much of the region.

Our analysis was less able to shed light on how indirect socioeconomic drivers have shaped patterns of biodiversity loss. Despite the suggestion that forest loss and degradation tend to be concentrated in poorer countries^48^, there was no clear pattern between trends of BII and GDP over time, and only a tenuous relationship between temporal trends in BII and GDP per capita in 2001. It is possible that the effect of GDP changes on BII may be masked in our data because of limitations in the treatment of plantation forest. Higher GDP can lead to lower biodiversity through increased investment into cropland and thus increased rates of forest conversion to plantations^49^. While we attempted to include the impact of plantation forests in our analysis by considering the effects similar to more intensively-used secondary vegetation (see “Methods” for more detail), if the impact of plantation forests are not adequately included in projections of biodiversity change, correlations with GDP may be muted.

The average BII values reported here are substantially lower than our earlier global estimate, where BII across the terrestrial surface was found to be approximately 84.6%, with tropical forest biomes estimated to range from 86% (tropical and subtropical dry broadleaf forests) up to 93% (for tropical and subtropical moist broadleaf forests) (with an average across the tropical and subtropical forest biomes of 92%)^35^. Six factors contribute to this difference. First, the new land-use maps have stricter bounds on the extent of primary forest. Second, we have fitted a model using data from tropical and subtropical forest biomes only, recognising that there are often regional differences in response to human impacts, both in terms of alpha^42,66^ and beta diversity^67^. Third, we compare biodiversity to a baseline of minimally-used primary vegetation rather than to the less stringent baseline of all primary vegetation used previously^35^, meaning that we now more closely approximate the idealised reference condition^21^. Fourth, our models of compositional similarity use data more efficiently than previously. Our earlier models compared each site with at most one other site to avoid pseudoreplication, which led to some sites being discarded and greatly limited the complexity of models that could be fitted; the models used here instead compare each site with each other site within the same study, using permutation tests to avoid elevated Type I error rates, and the richer data set means that we are able to account for additional habitat degradation related to roads and human population density in these models^68^. Fifth, we now use logit rather than log transformation of compositional similarity, which is more appropriate given that compositional similarity is continuous and bounded between zero and one. Sixth, we have attempted here to incorporate into projections the impact of plantation forests (by assuming that plantation forest is most likely to be incorporated into lightly- and intensively-used secondary forest in the land-use maps), which can drive major biodiversity loss, particularly when expanding at the expense of primary forest^42,66,69^.

We have provided a method for estimating annual change in BII in the hope that this global indicator of local terrestrial biodiversity can better inform policy at national and international levels by highlighting key areas for conservation or restoration and monitoring progress towards conservation and restoration targets. BII, as a measure of biotic integrity, is one of three suggested indicators in a post-2020 biodiversity framework aiming to “bend the curve” of biodiversity loss, with targets proposed for the proportion of biomes and ecoregions that should be within the planetary boundary by 2030 and 2050^29^. Although no national or global biodiversity commitments have yet been specified using BII, such model-derived indicators offer two potential advantages over indicators based on biodiversity time-series. First, because remote-sensing can detect land-use change in near-real time, they can potentially overcome the reporting lag that arises when compiling and synthesising biodiversity time series into an indicator (e.g., about four years with the Living Planet Index^70^). Second, the pressure-response framework in principle makes it easier to assess whether the sum of proposed actions to tackle drivers (such as nationally defined contributions) will be sufficient to achieve the desired state^71^. Such models, combined with targeted monitoring to validate or refine their projected trajectories, can enable timely adjustments to policy responses if needed. This feature—important in the climate arena—was lacking in the Aichi Targets, which were all missed^72^ despite a mid-term assessment^1^ having warned that they would be. BII effectively converts measured anthropogenic pressures into estimated biodiversity consequences based on statistical modelling of relevant biodiversity data sets. It therefore attempts to go beyond composite indicators of total anthropogenic pressure such as the Human Footprint Index^73^. The significant interactions among pressures in our models highlight a limitation of composite indicators (which implicitly assume effects are additive) but also complicate disaggregation into a simple understanding of responses to different pressures.

While our approach has dealt with many of the previous issues with estimating and projecting BII^74,75^, there are a number of caveats to our approach that must be considered as well as limits to the interpretation of results.

### Limitations and future work

There are two conceptual reasons why our implementation of BII may still underestimate biodiversity loss to date. Firstly, the compositional similarity metric implied in the original definition^21^ is permissive, in that the species abundance distribution could be completely reorganised without reducing BII, provided that the total abundance of originally-present species is not reduced and novel species are not introduced. Therefore, a region with high BII can still under some circumstances have shown strong losses in other aspects of diversity^76^. Using a combination of beta-diversity metrics may provide a more comprehensive assessment of the state of diversity^31,32^ and is a natural avenue for future development of BII. Secondly, we have so far only considered the impacts of land-use change and related pressures (human population and road density). Although these are the most important drivers of biodiversity loss in the recent past and near future, particularly in the tropics^77^, climate change is likely to become increasingly important over longer timescales^78^, especially as forest conversion already leads to strong changes in local temperature, potentially exacerbating future impacts of climatic change^79^. Incorporating both land-use change and climate change impacts is likely to improve estimates of biodiversity change^78,80,81^.

The biodiversity data come from spatial rather than temporal comparisons^19^. This means that we assume that either there are no lags in response or the data are sampled after communities have again reached equilibrium^19,82,83^; the former is exceptionally unlikely^82^ and the latter is unlikely to be true for all studies in the dataset. The result of these assumptions may lead to us mis-estimating the response of biodiversity to land-use change. However, spatial studies are more easily conducted—and therefore collated—than temporal studies; our overall dataset is therefore less hindered by geographic and taxonomic biases than any temporal dataset currently available^2,61,83^. In addition, temporal datasets rarely link biodiversity to specific pressures, making it difficult to extrapolate across space and time under using information on pressures. The spatial dataset that underlies our models still has gaps in coverage, but because the biodiversity data is linked to pressures, we can extrapolate responses to ‘fill in the gaps’. Such extrapolations become less reliable if responses vary across regions—limiting our analyses to data for tropical and subtropical biomes therefore makes the extrapolations more reliable^84^.

Additionally, the data we have used for anthropogenic pressures in our spatial and temporal projections vary in their resolution and in their accuracy. This is particularly important for road networks, which can grow rapidly; however, the data are only available as a static layer, and the completeness varies regionally. Static road layers may still provide insights into biodiversity responses: for instance, roads built pre-2000 were associated with forest loss in the following decade in Borneo^85^. However, other linear infrastructure, such as gas lines, can also have important consequences for biodiversity that are not included here^86^. Human population density was interpolated between time steps and, as the data are downscaled using an areal-weighting approach, its resolution at the pixel level varies depending on the size of the input areal unit^87^. Our models also assume that the response of biodiversity to land-use change is immediate; lagged responses are likely to be common and may be complex^19,82,83^. These difficulties mean that, while we have estimated BII at a 30" resolution, the estimates are most suitable for assessing average changes across larger pixels or areas (e.g., at a country level) and across broader time steps, rather than focussing on pixel-by-pixel and year-on-year changes.

In common with many other widely-used biodiversity indicators^88^, BII has not yet been sufficiently thoroughly evaluated to be sure what level of inferred change is needed to give confidence of real change on the ground given the imperfections and uncertainties in data and models. However, it is clear that estimates of change will be more uncertain for smaller areas. Appendix Fig. 2 plots the log response ratio of BII ${ln}{(BII)}_{2012}/{(BII)}_{2001}$ against *log10*(country area (km^2^)), showing the 0.025%, 50% and 97.5% quantile regressions; while there is not a strong relationship, the figure highlights countries that, for their size, have experienced unusually rapid BII change. An important step for future work would be to incorporate parameter uncertainty in both the abundance and compositional similarity models to provide uncertainty bounds on estimates and trends. This will be important especially for urban areas, where the data used here are limited and so projected diversity is more uncertain. This effort is made both more complicated and more important by the fact that BII is based upon two statistical models rather than one. One option is to take samples from the standard errors of the model estimates to produce a range of possible projections; cross-validation provides another possible route. A thorough exploration of error propagation is beyond the scope of the present study. However, future work could aim to incorporate uncertainty estimates both from the biodiversity models and the driver data.

Validation of BII would ideally come from comparing model outputs with observational data to assess the model skill^89^, rather than from measures of model fit such as R^2^ (as has been done for the underlying land-use data^90^). However, the broad taxonomic and ecological spread of the data used in modelling BII presents a major challenge for such an evaluation: part of the original motivation behind BII was precisely the lack of time-series data covering a broad range of taxa^19,21^. Clade-specific responses to anthropogenic pressures, which average out in the modelling because of the taxonomic representativeness of the PREDICTS database^19^, preclude using observed trends for particular taxa to test hindcasts. Robust assessment of skill is an important future challenge for many ecological models^91^: no global biodiversity indicators have yet been tested thoroughly in this way^92^.

Although estimating how BII has changed across space and time includes many underlying assumptions and uncertainties, the approach we have used goes far beyond our previous implementation of BII by producing annual estimates based on improved statistical modelling and time-varying data on land-use change derived from remote sensing. These annual estimates provide a useful tool for policy makers hoping to track progress towards national and international targets, and for assessing the state of nature; they also provide further evidence of the perilous state of tropical forest biodiversity^93^.

## Methods

Spatial and temporal projections of BII are produced by multiplying together the projections from statistical models of the two components of BII: overall organismal abundance (relative to overall abundance in the reference condition) and compositional similarity to an intact assemblage^94^. We therefore first describe the statistical models relating site-level biodiversity to anthropogenic pressures (land-use change, human population density and road density), then how these models were projected, and finally how the resulting BII estimates were analysed at the national and regional level.

### Statistical models of how biodiversity responds to anthropogenic pressures

#### Biodiversity data

Biodiversity data came from the PREDICTS database, a global collation of spatial comparisons^36^. The database contains surveys (‘studies’) of multiple sites differing in land use and related pressures^95^. The data were subset to only those sites in the following tropical or subtropical forested biomes: tropical and subtropical coniferous forests, tropical and subtropical dry broadleaf forests and tropical and subtropical moist broadleaf forests^96^. Although the PREDICTS database is somewhat biased towards north temperate latitudes, under-represented biomes were specifically targeted during its compilation, so it has reasonable coverage of tropical and subtropical regions^36^. We only used studies that sampled communities, excluding studies that focused on single species. All sites included had known geographic coordinates (so that the geographic distance among sites in a study could be calculated). Where enough information was provided in the methods of the original paper, for each site the sampling grain was estimated as the ‘maximum linear extent’; for example, the total transect length walked when sampling at a site^95^. The final dataset used for analyses contained 777,173 records from 180 published sources on the abundance of 20,740 species from 5159 sites worldwide (representing 45 countries; see Appendix Fig. 3 for a map of sites). Invertebrates make up 42.9% of the species, plants 36%, vertebrates 18.7% and fungi 2.4%.

#### Anthropogenic pressure data

The PREDICTS database holds site-level data on land use (primary vegetation, secondary vegetation, plantation forest, cropland, pasture or urban) and land-use intensity (minimal, light and intense), classified using information in the original sources or provided by their authors^95^. Although plantation forest exists as a separate land-use class in the PREDICTS database and is characterised by assemblages that are both relatively low in species richness and compositionally distinct^66,97^, it is rarely separated from other forests in global land-use layers; this is also true here. One option^35^ is to model responses to plantation forest but omit the effect when projecting results across space. Given the importance of plantation forests in tropical forested areas, we chose instead to group plantation forests together with secondary vegetation when modelling. We did this because it is the most likely source of plantation forest in the global land-use layers and because previous pan-tropical analyses have shown little difference between losses caused by primary conversion to secondary vegetation or plantation forest^42^, unless the plantations are intensive^66^. Lightly- and intensively-used plantation were therefore included with intensively-used secondary vegetation, and minimally-used plantation was included with lightly-used secondary vegetation. In addition to land-use and intensity, we included as pressures human population density^98^ and the density of roads^99^; we estimated the latter at two spatial scales, using their values within both the 1-km and 50-km grid cells containing each site. Compositional similarity between two sites is expected to reflect their environmental similarity as well their geographic proximity and the anthropogenic pressures that they face. We therefore extracted environmental conditions for each site from WorldClim (elevation, maximum temperature of the warmest month, minimum temperature of the coldest month, precipitation of the wettest and driest month^100^).

#### Mixed-effects models

Two mixed-effects models^101^ were run. The first model focussed on total abundance of organisms, calculated as the sum of abundance across all species recorded at each site. If sampling effort varied among sites within a study, and abundance was reported in an effort-sensitive metric (e.g., count of individuals), abundance was divided by sampling effort to make the numbers directly comparable^84^. Within each study, total abundance was then rescaled so that the maximum value was unity; this rescaling reduces the inter-study variance caused by differences in sampling effort and taxonomic focus and so facilitates modelling. Rescaled total abundance was square-root transformed prior to modelling, which used Gaussian errors; non-integer abundances in the original data precluded modelling of untransformed values with Poisson errors, and square-root transformation resulted in a better residual distribution than *ln*-transformation. Rescaled total abundance was modelled as a function of the following fixed effects: site-level land use and intensity (LUI), human population density ($ln(x + 1)$ transformed), and density of roads at the 1 km and 50 km scale (cube-root transformed), along with two-way interactions of LUI with each other anthropogenic pressure. We included an additional control variable to account for among-study differences in human population density (by taking the mean value within each study); this was to control for potential sampling and detection biases where sampling may be more complete in areas of higher human population density (which are generally closer to research institutions and more accessible for sampling). All continuous explanatory variables were standardised (centered and scaled to give a mean of zero and standard deviation of one) to reduce collinearity. We used a random-effect structure of spatial block within study, to account for differences in sampling methodology and large-scale environmental differences across studies and the spatial structure of sites within studies. With the model fitted using Restricted Maximum Likelihood (REML), we assessed whether random slopes were required by comparing Akaike’s Information Criterion (AIC) for models with each variable fitted as a random slope in turn. The best fixed-effects structure was then determined using backwards stepwise model simplification with the model fit using Maximum Likelihood^102^. Bootstrapping was used to estimate significance of coefficient values in the final model.

The second model assessed the response of compositional similarity to human impacts. We excluded studies where sampling effort varied among sites. For studies with at least one site classed as minimally-used primary vegetation (the baseline site), we calculated for each study in turn the compositional similarity of each site to each baseline site, measured as the proportion of site *j*’s individuals that belong to species also present in site *i* (where site *i* is in minimally-used primary vegetation, i.e., an asymmetric version of the abundance-based Jaccard similarity index^103^). Compositional similarity was logit transformed (car package, version 2.1-6^104^; an adjustment of 0.01 was used to account for values of 0 and 1). Compositional similarity between any pair of sites will be influenced by how much more impacted site *j* is than the baseline site *i*, as well as the absolute level of pressure faced by site *j*. For each continuous pressure variable, we therefore include in the models both the value at site *j* as well as the difference in value between site *i* and site *j*. We included geographic distance (*ln*-transformed) and environmental distance calculated as Gower’s dissimilarity^105^ using the gower package in R^106^, (cube-root transformed) between sites to account for decays in compositional similarity with distance^67^. Geographic distance was divided by the median maximum linear extent in the dataset prior to *ln*-transformation. The land-use contrast was included as a fixed effect along with its interactions with the continuous variables. As this dataset is more restricted than that used for abundance (because only studies that sample minimally-used primary vegetation can be used), we were not able to consider effects of use intensity within land uses, other than for primary vegetation (split into minimally-used primary vegetation and a combined class of lightly- and intensively-used primary vegetation) and secondary vegetation. Finally, we included the mean value of human population density within each study as a control variable. We included Study as a random intercept and assessed whether a random slope was supported by using the same framework as before, choosing the random structure with the lowest AIC value among the models that converged successfully. Backwards stepwise model simplification was performed to simplify the fixed effects structure of the model fit using Maximum Likelihood. Traditional significance tests based on likelihood ratios are not accurate here, because the data used are not independent (as each site is compared to multiple other sites within the same study). We therefore used permutations to determine whether a variable could be excluded from the model without significant loss of explanatory power^107^. We permuted the dataset 1000 times by randomly shuffling compositional similarity measurements within each study and refitting both the full and simplified model with this dataset. We then compared the likelihood ratio of our observed models with the distribution of likelihood ratios from models using the 1000 permuted datasets to assess whether the ratio was significantly higher than expected based on models with the same differences in parameters. We used a similar approach to estimate the significance of coefficient values in the final model. Note that this approach to modelling compositional similarity makes fuller use of the data than that used in a previous analysis^35^, which compared independent pairs of sites within studies and averaged coefficients across 100 models fitted to different randomly-chosen sets of pairwise comparisons. Our matrix-based approach uses all relevant site comparisons in the same model, allowing us to estimate more fixed effects, but carries with it the need for permutation tests to assess significance of variables and coefficients^107^.

Diversity analyses were performed using R Statistical Software^108^ version 3.4.3. Prior to modelling, all explanatory variables were assessed for multicollinearity using Generalized Variance Inflation Factors^109^ for each model; all values were below 5, indicating acceptable levels of collinearity. Transformation of explanatory variables were chosen based on improvements to residual distribution.

### Global anthropogenic pressure data and maps of BII for each year

#### Land use

Hoskins et al.^90^ statistically downscaled global land-use data for the year 2005 from 0.5-degree resolution^110^ to 30 arc-second resolution, estimating the fraction of each pixel in each of the following classes: primary habitat, secondary habitat, cropland, pasture and urban. That approach was extended here, by integrating the static data for 2005 with remotely-sensed time-varying data on land cover and forest change. The original method described in Hoskins et al.^90^ uses a combination of Generalised Additive Models (GAMs) and constrained optimisation to produce fine-grained predictions of multiple land-use classes using the best-available spatial data on climate, landform, soil, land cover, human population density and accessibility (at 30 arc second resolution) as inputs (See Appendix Table 1 for details). The downscaled land-use maps produced by this method were validated in a number of ways, including against the original coarser-scale land-use data^110^ and against an independent dataset of land use (the PREDICTS database, which includes land-use classes for sites within the dataset)^90^. While there is of course still uncertainty in the downscaling models, the validation showed this producedure to be effective^90^.

We made several modifications to this method in order to generate our land-use time-series. To improve predictions outside of the fitted parameter space, we performed AIC-based backwards stepwise model selection, to identify the most parsimonious set of predictor variables. We then fitted our downscaling models to the year 2005 coarse-grained Land-Use Harmonisation data^110^ and, using time-varying covariates, used these models to predict land use for the full time-series. Our time-varying covariates were derived from Collection 5 MODIS Global Land Cover Type product, which has a yearly temporal resolution^111^. Once our downscaling models were fitted to the 2005 data of this land-cover dataset, we were able to predict land-use change using the remaining years in the time-series.

We maximised the influence of the time-varying covariates in our downscaling models by fitting the GAMs in two stages. Initially the GAMs were fitted to only the time-varying covariates (i.e., annual land-cover datasets), allowing these to explain as much variation in the data as possible. The static covariates were fitted only in a second step, so that they were only able to describe variation not already described by the time-varying covariates. This resulted in models that maximised information coming from the time-varying land-cover data and, as such, reflected the temporal change in the land-cover layers as much as possible in our land-use predictions. Within tropical and sub-tropical forested regions (defined as Tropical and Subtropical Moist Broadleaf Forests, Tropical and Subtropical Dry Broadleaf Forests and Tropical and Subtropical Coniferous forests in^96^), we further refined our land-use estimates by integrating the Global Forest Changes (GFC) dataset^44^ using the following rules. Within a cell, when the predicted proportion of primary habitat was greater than observed by GFC, primary habitat was reduced to match the GFC- observed forest cover. All other land uses were then scaled proportionally to their predicted values to ensure all constraints were met. When the sum of predicted primary and secondary habitat were less than observed GFC data they were scaled proportionally so that their sum matched the GFC data. The remaining three land uses were then scaled proportionally to ensure all constraints were met. This provided land-use estimates within forest biomes that were consistent with the observed change in the GFC dataset. Note that this procedure can result in occasional increases in the amount of primary vegetation over time.

#### Human population density

We downloaded human population density data for the years 2000, 2005, 2010 and 2015 from^98^ (adjusted to match 2015 revision of UN WPP Country Totals). After $ln(x + 1)$ transformation (the 1 is added to avoid problems caused by zeros in the data), we interpolated data for intervening years by assuming linear change in the *ln*-transformed value over time (i.e., assuming that populations grow exponentially^98,112^). For example, a cell’s value for 2006 is given by $0.8 \times$ value for $2005+ 0.2 \times$ value for 2010.

#### Density of roads

We used a vector map of the world’s roads^99^ to derive maps of road density: for each 30 arc-second cell, road length is calculated within a 1km and 50km radius from the centre point of the cell and expressed as density per 30 arc-second cell (approximately 1km^2^) of land (using the arcpy functions of LineLength and FocalStatistics, ArcGIS v10.5). In the absence of any global time-series data of roads, we treated this layer as a static, rather than dynamic, pressure in our projections. However, it should be noted that there are still substantial gaps in the gRoads dataset, particularly in South and East Asia^113^.

#### Land-use intensity

To estimate land-use intensity for each year, we applied the statistical models of^97^ of land-use intensity to each year’s data on land use and human population density. Briefly, Newbold et al.^97^ reclassified the Global Land Systems dataset^114^ into land-use/use-intensity combinations and then modelled how the proportional coverage of each combination within each $0.5^{\circ }$ grid cell depended on the proportion of the grid cell under that land use, human population density and UN sub-region (and all two- and three-way interactions).

#### Maps of modelled BII for each year

We used each year’s maps of land use, land-use intensity and human population density, along with the (static) maps of road density to drive the two statistical models of how biodiversity responds to anthropogenic pressures. Anthropogenic pressure data were not permitted to exceed the ranges found among sites in the biodiversity dataset (the values were capped), to prevent extrapolation beyond our data.

For total abundance, the modelled responses were back-transformed (squared) and expressed relative to the modelled estimate for the baseline condition of minimally-used Primary vegetation with zero human population and road density (Note that the baseline estimate comes from the statistical model (predictions) rather than from just combining data from sites where all anthropogenic pressures match the baseline conditions.) For compositional similarity, values were back-transformed (inverse-logit with adjustment) and expressed relative to the modelled estimate for the baseline, i.e., the compositional similarity between two minimally-used Primary vegetation sites, both with zero human population and road density, having the same environment (zero environmental distance between the sites) and being adjacent (geographic distance between them equating to the median sampling grain in the dataset). Control variables (study-level mean values of human population density and environmental variables) were set to zero for this step. We multiplied the spatial projections of overall abundance and compositional similarity together to estimate BII. We did this for each year between 2001 and 2012.

Average BII values for each country, subregion and region were calculated for each year by averaging modelled values across all grid cells intersecting the relevant region’s shape file (as defined for the IPBES assessment, from^115^) after re-projecting to a Behrmann equal-area projection. To assess overall trends across the time period, we calculated the log response ratio of start (year 2001) and final (year 2012) values as ${ln}({BII}_{2012}/{BII}_{2001})$. Wilcoxon signed-rank tests were used to assess average trends across all countries. We also relate these changes to contemporaneous changes in GDP per capita (in current US dollar^116^) and GDP levels at the start of the time series ($log10-$transformed value at 2001). We ran linear mixed effects models^117^, including IPBES subregion as a random intercept to account for spatial autocorrelation among neighbouring countries. Simulated model residuals were also tested for spatial autocorrelation. For models including the log response ratio of GDP as an explanatory variable, spatial autocorrelation was still evident in the residuals^118–120^, so a gaussian spatial autocorrelation structure was included in the model^117^. Models were run on all countries and on only those where at least 50% of their area was included in the projections; results did not vary qualitatively so we report results for all countries.

## Supplementary information

Supplementary Information 1.

## Data Availability

The biodiversity used here are openly available for download from the NHM data portal (data.nhm.ac.uk) along with summary statistics for land use and BII for each country and region (10.5519/5wriutqz). Summary statistics are also available from the Biodiversity Indicators Partnership portal (bipdashboard.natureserve.org). The land-use layers are openly available on the CSIRO Data Access Portal.
