# Supplementary material for: Annual changes in the Biodiversity Intactness Index in tropical and subtropical forest biomes, 2001–2012
Source: Sci Rep. 2021 Oct 12;11:20249. doi: 10.1038/s41598-021-98811-1 (PMC8511124; doi:10.1038/s41598-021-98811-1)
Supplement: Supplementary file 1 — Supplementary Information 1. [file 41598_2021_98811_MOESM1_ESM.pdf]

Annual changes in the Biodiversity Intactness Index in tropical and subtropical forest biomes, 2001-2012: Supplementary Material 1

Adriana De Palma[1], Andrew Hoskins[2, 3], Ricardo E. Gonzalez[4], Luca Börger[5], Tim Newbold[6], Katia Sanchez-Ortiz[1, 4], Simon Ferrier[2], and Andy Purvis[1, 4]

[1]Natural History Museum, Department of Life Sciences, London, SW7 5BD, UK
[2]CSIRO Land and Water, Canberra, ACT, Australia
[3]CSIRO Health and Biosecurity, Townsville, Qld, Australia
[4]Imperial College London, Department of Life Sciences, London, SL5 7PY, UK
[5]Department of Biosciences, University of Swansea, Swansea, SA2 8PP, UK
[6]Centre for Biodiversity and Environment Research, Department of Genetics, Evolution and Environment, University College London, Gower Street, London WC1E 6BT, UK

August 28, 2021

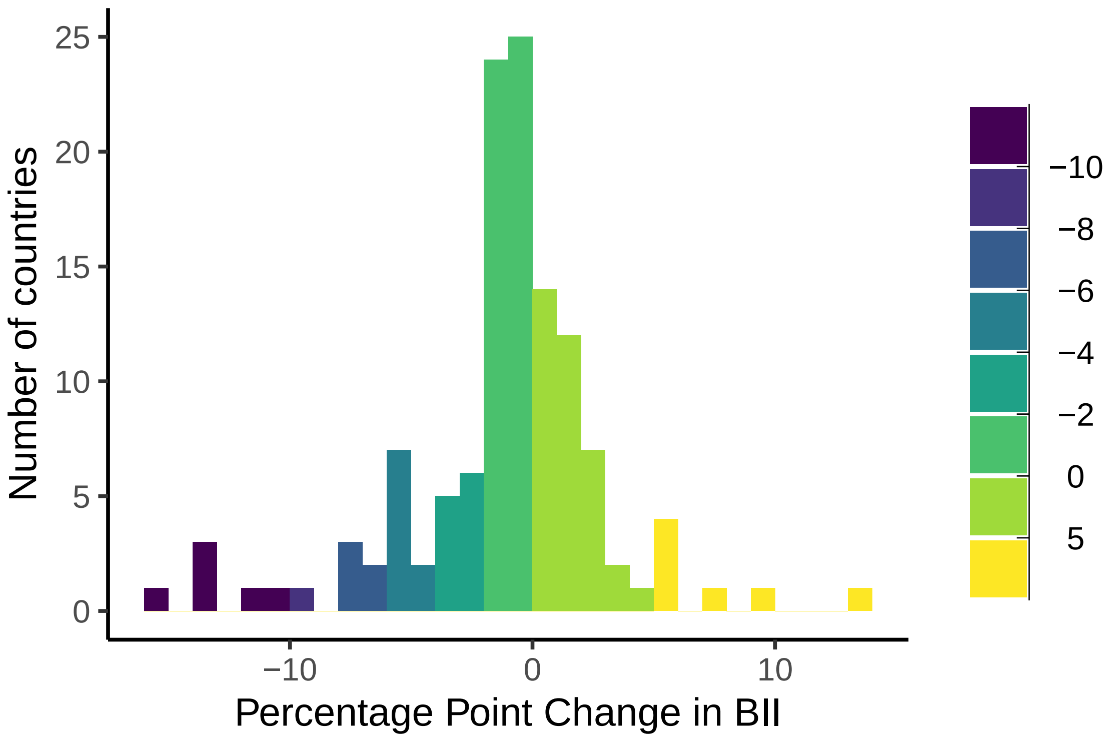

Appendix Figure 1: Distribution of country-level averages of change in BII over time. Change was calculated as the percentage point change between the average BII value for 2001 and 2012 for a given country. A value of zero indicates no change, negative values indicate a decline from 2001 to 2012, and positive values indicate an increase between 2001 and 2012. The colour scheme matches Figure 1 in the main manuscript.

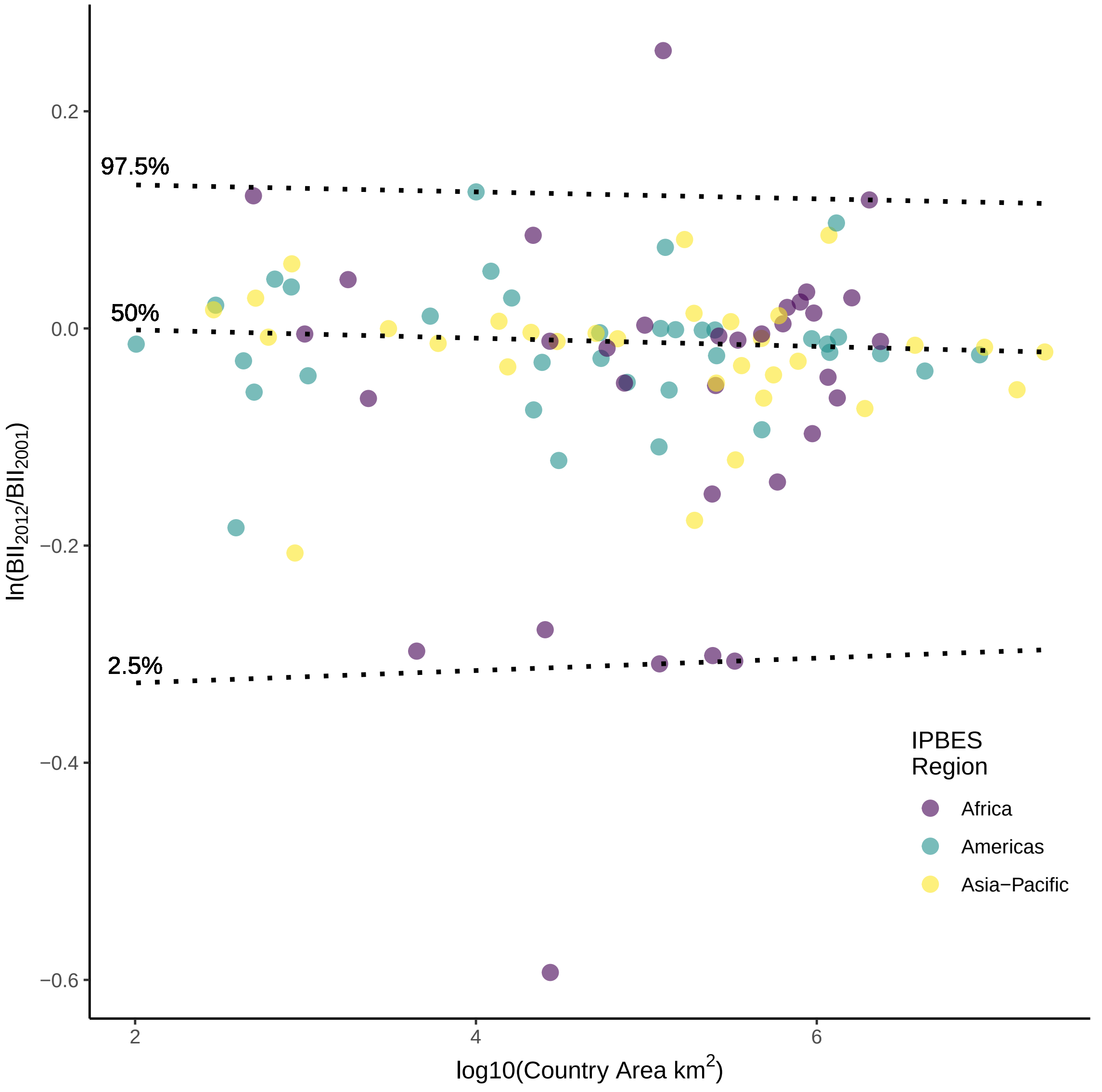

Appendix Figure 2: Change in BII over time plotted against the area of each country. Change was calculated as the log-response ratio of 2012 and 2001 values. A value of zero indicates no change, negative values indicate a decline from 2001 to 2012, and positive values indicate an increase between 2001 and 2012. Quantile regression lines are shown; points outside the top and bottom lines indicate countries with changes in BII that are more extreme than expected given the country area.

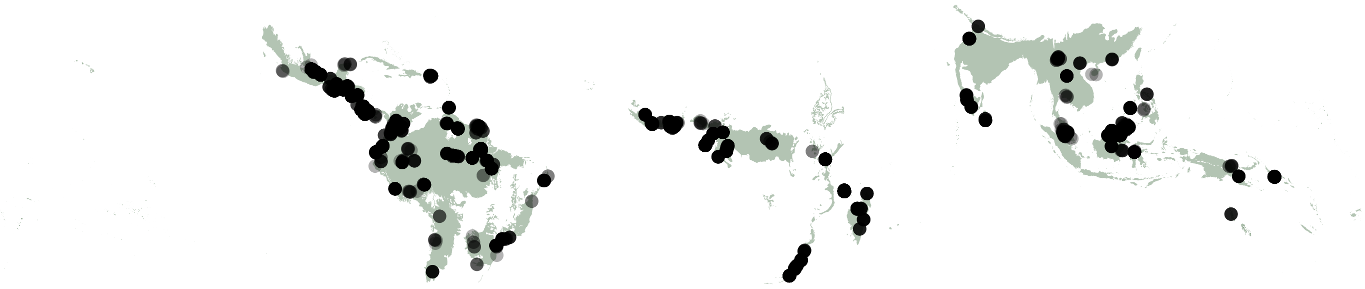

Appendix Figure 3: Sites from the PREDICTS database included in the modelling. Tropical and subtropical forests are shown in light green. Sites are displayed as semi-transparent black points (darker points therefore indicate where multiple sites are in close proximity).

Appendix Table 1: Data layers used as covariates or masking layers during the downscaling process to create the land-use maps. This table has been reproduced from Hoskins et al. (2016).

**Description**	**Reference**
MOD16 dataset gap filled with Annual Actual Evaporations calculated as the sum of monthly EA derived using the Budkyo framework based on WorldClim climatic data, using PAWHC calculated from 1 km Soil Depth from www.soilgrids.org combined with AWC from theHarmonized World Soil Database	Hijmans et al. (2005); Mu et al. (2007); FAO/IIASA/ISRIC/ISSCAS/JRC (2012); Hengl et al. (2014)
Mean Annual Temperature with maximum and minimum temperature corrected for radiation differences due to variation in terrain based on Danielson and Dean (2011) following Wilson and Gallant (2000)	Wilson and Gallant (2000); Hijmans et al. (2005); Danielson and Dean (2011)
Annual precipitation. Sum of monthly precipitation from WorldClim	Hijmans et al. (2005)
Topographic Wetness Index. Calculated at 9 and upscaled to 30	Reuter and Hengl (2012)
Presence of permanent ice	Olson et al. (2001)
Slope calculated at 9 and upscaled to 30	Reuter and Hengl (2012)
Soil Organic Carbon content. Weighted average of all depth classes	Hengl et al. (2014)
Presence of permanent water bodies	Lehner and Döll (2004)
Global Accessibility Index. The travel time to the nearestpopulation center of 50,000 or more	Uchida and Nelson (2009)
Population density	Balk et al. (2005)
Consensus land cover. 30 land-cover product made byharmonizing multiple products	Tuanmu and Jetz (2014)

Appendix Table 2: Coefficients from the mixed effects model of total abundance. Asterisks identify estimates that are statistically significant (where confidence intervals did not cross zero). Confidence intervals were assessed using bootstrapping. The overall R^2 for this model was 0.67 Xu (2003); Ldecke et al. (2021). The marginal and conditional R^2 for this model was 0.05 and 0.65 respectively Barton (2020); Nakagawa and Schielzeth (2013). Note that given the high levels of variability among studies, we expect the random effects to explain far more variation than fixed effects in any given model.

Term	Coefficient	Estimate	Standard Error	t-statistic	Lower 95% Confidence Interval	Upper 95% Confidence Interval
Land Use and Intensity (LUI)	(Intercept)	0.6945 *	0.02	41.45	0.66	0.73
	Primary Vegetation Light use	-0.0033	0.01	-0.22	-0.03	0.02
	Primary Vegetation Intense use	-0.0246	0.03	-0.97	-0.07	0.03
	Secondary Vegetation Minimal use	-0.0303	0.02	-1.71	-0.07	0.00
	Secondary Vegetation Light use	-0.001	0.02	-0.06	-0.04	0.03
	Secondary Vegetation Intense use	-0.064 *	0.02	-3.51	-0.10	-0.03
	Cropland Minimal use	-0.0478	0.04	-1.26	-0.13	0.03
	Cropland LightIntense	-0.1032 *	0.04	-2.67	-0.18	-0.02
	Pasture Minimal use	-0.1715 *	0.05	-3.61	-0.27	-0.07

Term	Coefficient	Estimate	Standard Error	t-statistic	Lower 95% Confidence Interval	Upper 95% Confidence Interval
	Pasture LightIntense	-0.0901 *	0.04	-2.26	-0.17	-0.01
	Urban	-0.0106	0.07	-0.15	-0.17	0.13
Human Population Density (HPD)		0.0141	0.01	1.11	-0.01	0.04
Road Density (50km)		-0.0283	0.02	-1.81	-0.06	0.00
Study-level mean Human Population Density		0.0027	0.02	0.17	-0.03	0.04
LUI:HPD	Primary Vegetation Light use : HPD	0.0081	0.01	0.59	-0.02	0.03
	Primary Vegetation Intense use : HPD	0.0151	0.03	0.52	-0.04	0.07
	Secondary Vegetation Minimal use : HPD	-0.0422 *	0.02	-2.57	-0.08	-0.01
	Secondary Vegetation Light use : HPD	-0.0362 *	0.02	-2.10	-0.07	-0.00
	Secondary Vegetation Intense use : HPD	-0.0043	0.02	-0.24	-0.04	0.03

Term	Coefficient	Estimate	Standard Error	t-statistic	Lower 95% Confidence Interval	Upper 95% Confidence Interval
	Cropland Minimal use : HPD	-0.0343	0.05	-0.75	-0.14	0.06
	Cropland LightIntense : HPD	-0.0318	0.02	-1.64	-0.07	0.00
	Pasture Minimal use : HPD	0.0895	0.05	1.63	-0.04	0.20
	Pasture LightIntense : HPD	0.0157	0.03	0.47	-0.05	0.08
	Urban : HPD	0.0334	0.03	1.09	-0.03	0.10
LUI:Road Density (50km)	Primary Vegetation Light use : Road Density (50km)	-0.0419 *	0.02	-2.55	-0.07	-0.01
	Primary Vegetation Intense use : Road Density (50km)	-0.0292	0.05	-0.60	-0.12	0.06
	Secondary Vegetation Minimal use : Road Density (50km)	0.0145	0.02	0.82	-0.02	0.05
	Secondary Vegetation Light use : Road Density (50km)	0.0124	0.02	0.63	-0.03	0.05

Term	Coefficient	Estimate	Standard Error	t-statistic	Lower 95% Confidence Interval	Upper 95% Confidence Interval
	Secondary Vegetation Intense use : Road Density (50km)	-0.0062	0.02	-0.31	-0.05	0.03
	Cropland Minimal use : Road Density (50km)	0.1027 *	0.03	3.20	0.04	0.17
	Cropland LightIntense : Road Density (50km)	-0.0153	0.05	-0.34	-0.11	0.07
	Pasture Minimal use : Road Density (50km)	0.0714	0.04	1.92	-0.01	0.15
	Pasture Light-Intense : Road Density (50km)	0.0065	0.04	0.17	-0.07	0.09
	Urban : Road Density (50km)	-0.0585	0.05	-1.26	-0.15	0.04

Appendix Table 3: Coefficients from the mixed effects model of compositional similarity. Asterisks identify where the estimate was significantly different from estimates derived from randomised data (1000 randomisations) (p ¡ 0.05*, p ¡ 0.01**, p ¡ 0.001***). The overall R^2 for this model was 0.56 Xu (2003); Ldecke et al. (2021). The marginal and conditional R^2 for this model was 0.12 and 0.68 respectively Barton (2020); Nakagawa and Schielzeth (2013). Note that given the high levels of variability among studies, we expect the random effects to explain far more variation than fixed effects in any given model. The data used in models of compositional similarity are also pseudoreplicated; this may introduce a slight bias into R^2 values.

Term	Coefficient	Estimate	Standard Error	t-statistic
(Intercept)	(Intercept)	1.34 ***	0.24	5.69
Geographic Distance		-0.1745 ***	0.02	-11.24
Environmental Distance		-0.1946 ***	0.02	-11.13
Land Use and Intensity (LUI) Contrast	Primary Vegetation Minimal use-Cropland	-1.4972 ***	0.03	-45.78
	Primary Vegetation Minimal use-Pasture	-2.9842 ***	0.03	-86.72
	Primary Vegetation Minimal use-Primary Vegetation	-0.2277 ***	0.04	-5.63
	Primary Vegetation Minimal use-Secondary Vegetation	-0.6053 ***	0.02	-33.64
	Primary Vegetation Minimal use-Urban	-1.4562	1.56	-0.94
Human Population Density at comparator site (HPD)		0.3349 ***	0.04	9.45

Term	Coefficient	Estimate	Standard Error	t-statistic
Difference in Human Population Density between baseline and comparator site (HPD difference)		0.1351 ***	0.12	1.10
Road Density (1km) at comparator site (RD 1km)		-0.1624 ***	0.02	-8.61
Difference in Road Density (1km) between baseline and comparator site (RD 1km difference)		-0.047 **	0.02	-2.97
Road Density (50km) at comparator site (RD 50km)		-0.5185 ***	0.06	-8.91
Difference in Road Density (50km) between baseline and comparator site (RD 50km difference)		-0.0269	0.02	-1.75
Study-level mean Human Population Density		0.0586 ***	0.22	0.27
Geographic Distance : LUI Contrast	Geographic Distance : Primary Vegetation Minimal use-Cropland	0.0058	0.03	0.17
	Geographic Distance : Primary Vegetation Minimal use-Pasture	0.7683 ***	0.04	20.96

Term	Coefficient	Estimate	Standard Error	t-statistic
	Geographic Distance : Primary Vegetation Minimal use-Primary Vegetation	-0.1396 ***	0.03	-4.30
	Geographic Distance : Primary Vegetation Minimal use-Secondary Vegetation	0.0281	0.02	1.41
	Geographic Distance : Primary Vegetation Minimal use-Urban	-0.8726 *	0.46	-1.88
Environmental Distance : LUI Contrast	Environmental Distance : Primary Vegetation Minimal use-Cropland	0.0637 *	0.03	1.91
	Environmental Distance : Primary Vegetation Minimal use-Pasture	-0.2919 ***	0.04	-6.59
	Environmental Distance : Primary Vegetation Minimal use-Primary Vegetation	0.1584 **	0.05	3.25
	Environmental Distance : Primary Vegetation Minimal use-Secondary Vegetation	-0.003	0.02	-0.15
	Environmental Distance : Primary Vegetation Minimal use-Urban	0.7441	0.60	1.24
LUI Contrast : HPD	Primary Vegetation Minimal use-Cropland : HPD	-0.1926 ***	0.04	-4.69

Term	Coefficient	Estimate	Standard Error	t-statistic
	Primary Vegetation Minimal use-Pasture : HPD	-0.9912 ***	0.07	-14.79
	Primary Vegetation Minimal use-Primary Vegetation : HPD	-0.0127	0.05	-0.27
	Primary Vegetation Minimal use-Secondary Vegetation : HPD	-0.3403 ***	0.03	-11.53
	Primary Vegetation Minimal use-Urban : HPD	-0.3784	0.91	-0.42
LUI Contrast : RD 1km	Primary Vegetation Minimal use-Cropland : RD 1km	0.084	0.07	1.19
	Primary Vegetation Minimal use-Pasture : RD 1km	0.7816 ***	0.05	15.35
	Primary Vegetation Minimal use-Primary Vegetation : RD 1km	-0.1305 **	0.04	-3.39
	Primary Vegetation Minimal use-Secondary Vegetation : RD 1km	0.079 *	0.03	2.97
	Primary Vegetation Minimal use-Urban : RD 1km	-0.1672	0.67	-0.25
LUI Contrast : RD 50km	Primary Vegetation Minimal use-Cropland : RD 50km	0.05	0.05	0.91

Term	Coefficient	Estimate	Standard Error	t-statistic
	Primary Vegetation Minimal use-Pasture : RD 50km	0.8559 ***	0.06	13.62
	Primary Vegetation Minimal use-Primary Vegetation : RD 50km	-0.1694 ***	0.05	-3.66
	Primary Vegetation Minimal use-Secondary Vegetation : RD 50km	0.0556 **	0.03	2.05
	Primary Vegetation Minimal use-Urban : RD 50km	0.7592	0.80	0.95
LUI Contrast : HPD difference	Primary Vegetation Minimal use-Cropland : HPD difference	0.1972 ***	0.03	7.70
	Primary Vegetation Minimal use-Pasture : HPD difference	0.0014	0.04	0.04
	Primary Vegetation Minimal use-Primary Vegetation : HPD difference	-0.0865 *	0.04	-2.14
	Primary Vegetation Minimal use-Secondary Vegetation : HPD difference	-0.09 ***	0.02	-4.49
	Primary Vegetation Minimal use-Urban : HPD difference	0.0902	0.14	0.64
LUI Contrast : RD 1km difference	Primary Vegetation Minimal use-Cropland : RD 1km difference	-0.2287 **	0.08	-2.99

Term	Coefficient	Estimate	Standard Error	t-statistic
	Primary Vegetation Minimal use-Pasture : RD 1km difference	0.4563 ***	0.05	9.48
	Primary Vegetation Minimal use-Primary Vegetation : RD 1km difference	0.2671 ***	0.03	8.20
	Primary Vegetation Minimal use-Secondary Vegetation : RD 1km difference	-0.0271	0.03	-1.02
	Primary Vegetation Minimal use-Urban : RD 1km difference	-0.1598	0.34	-0.47
LUI Contrast : RD 50km difference	Primary Vegetation Minimal use-Cropland : RD 50km difference	0.1687 ***	0.05	3.35
	Primary Vegetation Minimal use-Pasture : RD 50km difference	-0.0088	0.04	-0.22
	Primary Vegetation Minimal use-Primary Vegetation : RD 50km difference	-0.028	0.03	-1.02
	Primary Vegetation Minimal use-Secondary Vegetation : RD 50km difference	-0.1187 ***	0.02	-5.26
	Primary Vegetation Minimal use-Urban : RD 50km difference	-1.2074 *	0.80	-1.52

References

D. Balk, F. Pozzi, G. Yetman, U. Deichmann, and A. Nelson. The distribution of people and the dimension of place: methodologies to improve the global estimation of urban extents. In *Urban Remote Sensing Conference*. International Society for Photogrammetry and Remote Sensing, Tempe, Arizona, 2005.

K. Barton. *MuMIn: Multi-Model Inference*, 2020. URL `https://CRAN.R-project.org/package=MuMIn`. R package version 1.43.17.

J. J. G. Danielson and B. Dean. Global multiresolution terrain elevation data 2010 (gmted2010): U.s. In *U.S.D.o.t. Interior, ed. Geological survey openfile report 20111073*, page 26. U.S. Geological Survey, Reston, VA., 2011.

FAO/IIASA/ISRIC/ISSCAS/JRC. Harmonized world soildatabase (version 1.2), 2012.

T. Hengl, J. M. de Jesus, R. A. MacMillan, N. H. Batjes, G. B. M. Heuvelink, E. Ribeiro, A. Samuel-Rosa, B. Kempen, J. G. B. Leenaars, M. G. Walsh, and M. R. Gonzalez. Soilgrids1km global soil information based on automated mapping. *PLOS ONE*, 9(8):1–17, 08 2014.

R. J. Hijmans, S. Cameron, J. Parra, P. Jones, and A. Jarvis. Very high resolution interpolated climate surfaces for global land areas. *Int. J. Climatol.*, 25:1965–1978, 2005.

A. J. Hoskins, A. Bush, J. Gilmore, T. Harwood, L. N. Hudson, C. Ware, K. J. Williams, and S. Ferrier. Downscaling land-use data to provide global 30 "estimates of five land-use classes. *Ecology and Evolution*, 6(9):3040–3055, 2016.

B. Lehner and P. Döll. Development and validation of a global database of lakes, reservoirs and wetlands. *Journal of hydrology*, 296(1-4):1–22, 2004.

D. Ldecke, M. S. Ben-Shachar, I. Patil, P. Waggoner, and D. Makowski. performance: An R package for assessment, comparison and testing of statistical models. *Journal of Open Source Software*, 6 (60):3139, 2021. doi: 10.21105/joss.03139.

Q. Mu, F. A. Heinsch, M. Zhao, and S. W. Running. .development of a global evapotranspiration algorithm based on modis and global meteorology data. *Remote Sens.Environ.*, 111:519–536, 2007.

S. Nakagawa and H. Schielzeth. A general and simple method for obtaining r2 from generalized linear mixed-effects models. *Methods in Ecology and Evolution*, 4 (2):133–142, 2013. doi: https://doi.org/10.1111/j.2041-210x.2012.00261.x. URL `https://besjournals.onlinelibrary.wiley.com/doi/abs/10.1111/j.2041-210x.2012.00261.x`.

D. M. Olson, E. Dinerstein, E. D. Wikramanayake, N. D. Burgess, G. V. Powell, E. C. Underwood, J. A. D'amico, I. Itoua, H. E. Strand, J. C. Morrison, et al. Terrestrial ecoregions of the world: A new map of life on earth: A new global map of terrestrial ecoregions provides an innovative tool for conserving biodiversity. *BioScience*, 51(11):933–938, 2001.

H. I. Reuter and T. Hengl. Worldgrids a public repository of global soil covariates. In *Digital soil assessments and beyond*, pages 287–292. Taylor Francis Group, Sydney, NSW, Australia, 2012.

M.-N. Tuanmu and W. Jetz. A global 1-km consensus land-cover product for biodiversity and ecosystem modelling. *Global Ecology and Biogeography*, 23(9):1031–1045, 2014.

H. Uchida and A. Nelson. Agglomeration index: Towards a new measure of urban concentration. *WIDER Working Paper*, 2009.

J. Wilson and J. Gallant. Secondary topographic attributes. In *Terrain analysis: principals and applications*, pages 51–85. John Wiley Sons, New York, NY, 2000.

R. Xu. Measuring explained variation in linear mixed effects models. *Statistics in Medicine*, 22(22):3527–3541, 2003. doi: https://doi.org/10.1002/sim.1572. URL https://onlinelibrary.wiley.com/doi/abs/10.1002/sim.1572.
